# Every Saturday

**Published:** 1868-12

**Authors:** 


EVERY SATURDAY.—The present number of "Every Saturday" contains the first of a series of papers, entitled "New Uncommercial Samples," by Mr. Chas. Dickens. These will be of the same general character as the popular "Uncommercial Traveler" papers, and may be regarded as a continuation of them.

The first of these is re-printed from advance sheets of Mr. Dickens's magazine, *All the Year Round*, and the succeeding papers will be promptly reproduced for the readers of *Every Saturday* in the same way.

